# The thymus medulla and its control of αβT cell development

**DOI:** 10.1007/s00281-020-00830-z

**Published:** 2020-12-11

**Authors:** Emilie J. Cosway, Kieran D. James, Beth Lucas, Graham Anderson, Andrea J. White

**Affiliations:** Institute of Immunology and Immunotherapy, Floor 4 Institute for Biomedical Research, Medical School, University of Birmingham, Birmingham, B15 2TT UK

**Keywords:** Thymus, T cell, Stromal cell, Thymocyte, Microenvironment

## Abstract

αβT cells are an essential component of effective immune responses. The heterogeneity that lies within them includes subsets that express diverse self-MHC-restricted αβT cell receptors, which can be further subdivided into CD4^+^ helper, CD8^+^ cytotoxic, and Foxp3^+^ regulatory T cells. In addition, αβT cells also include invariant natural killer T cells that are very limited in αβT cell receptor repertoire diversity and recognise non-polymorphic CD1d molecules that present lipid antigens. Importantly, all αβT cell sublineages are dependent upon the thymus as a shared site of their development. Ongoing research has examined how the thymus balances the intrathymic production of multiple αβT cell subsets to ensure correct formation and functioning of the peripheral immune system. Experiments in both wild-type and genetically modified mice have been essential in revealing complex cellular and molecular mechanisms that regulate thymus function. In particular, studies have demonstrated the diverse and critical role that the thymus medulla plays in shaping the peripheral T cell pool. In this review, we summarise current knowledge on functional properties of the thymus medulla that enable the thymus to support the production of diverse αβT cell types.

## Introduction

The thymus plays an essential role in the immune system by providing a unique environment to support αβT cell development. Unlike the bone marrow, the only other primary lymphoid organ, lymphocyte development in the thymus requires the continued importation of lymphoid progenitors to ensure that αβT cells are produced throughout life. As such, the ability of the thymus to recruit, foster, and export αβT cells effectively determines how the peripheral immune system mounts effective responses. It is now clear that the multi-stage process of αβT cell development requires serial interactions with stromal environments that form cortical and medullary area of the thymus. While early events in thymocyte development take place in the thymic cortex and are controlled by the cortical thymic epithelial cells (cTEC), the thymus medulla plays a pivotal role in events that ensure the correct formation of multiple αβT cell sublineages [1–3]. For example, for conventional αβT cell receptor (αβTCR) expressing thymocytes, the medulla purges the newly selected repertoire of autoreactive specificities via negative selection, and supports their post-selection thymocyte maturation and egress from the thymus [4]. In addition, through interactions with medullary thymic epithelial cells (mTEC) and dendritic cells (DC), the thymus medulla enables lineage diversion of CD4^+^ single-positive (CD4SP) thymocytes to Foxp3^+^ T regulatory (T-Reg) cells that control anti-self-immune responses [5]. Finally, the thymus medulla provides multiple signals that enable CD1d-restricted invariant natural killer T (iNKT) cells to complete their intrathymic maturation [6]. Thus, the thymus medulla generates multiple αβT cell types that play important roles in both innate and adaptive immunity. Indeed, the functional importance of the thymus medulla is readily evident in the disruption of immune homeostasis and manifestation of autoimmunity that occurs when its development and/or function is impaired. This can include the consequences of naturally occurring or experimentally induced genetic mutations that take place either in the cells that help form the medulla (e.g. Aire, Relb in mTEC) or in the cells that the medulla fosters (e.g. Foxp3 in T-Reg) [7–10]. Given this importance and the recent advances made in understanding the cellular complexity of mTEC, the primary focus of this review is to examine the cell types that reside within the thymus medulla, and examine how they form microenvironments that shape key events during intrathymic αβT cell development.

## mTEC functionality and function

### MHCII^hi^CD80^hi^ mTEC^hi^

mTEC are classically defined as an Ly51^−^UEA-1^+^ subset of total EpCAM^+^ TEC. Within the bulk mTEC population, analysis of MHCII and CD80 reveals mTEC^lo^ (MHCII^lo^CD80^lo^) and mTEC^hi^ (MHCII^hi^CD80^hi^) subsets. While much effort has been made to understand the developmental relationships of mTEC subsets, mTEC pathways are still not fully understood. For example, while mTEC^lo^ are known to contain progenitors of mTEC^hi^, they also contain cells that are ‘ex-mTEC^hi^’ and so represent late stages of mTEC development.

An important role of mTEC^hi^ is the induction of central tolerance, which involves the screening of αβTCR specificities that newly positively selected single positive (SP) thymocytes express. This becomes possible as a proportion of mTEC^hi^ express autoimmune regulator (Aire), with the development of Aire^+^ being driven by cell surface receptors that are members of the tumour necrosis factor superfamily, notably RANK [11–14]. Aire expression by mTEC^hi^ allows for the presentation of tissue-restricted antigens (TRAs) by via MHCI and MHCII, enabling the effective screening of developing thymocytes for their TCR specificity against self [11]. The molecular mechanisms that control Aire-mediated expression are beginning to emerge [15, 16]. Some studies propose that Aire functions through its recruitment to target genes where it causes localised histone modifications via either histone methylation or acetylation which relaxes chromatin for subsequent TRA transcription [17]. In addition, the protein deacetylase Sirtuin-1 or Sirt1 was shown to be highly expressed in mature Aire-expressing mTEC and it remains closely associated with Aire itself, resulting in the deacetylation of Aire that is necessary for its transcriptional activity [18]. In addition to Aire, some mTEC^hi^ express the transcription factor FEZ family zinc finger 2 (Fezf2), which has been reported to allow for expression of TRAs by mTEC that are distinct from those under the control of Aire [19]. However unlike Aire, Fezf2 is not restricted solely to mTEC^hi^, with Fezf2^+^ cells also present within the mTEC^lo^ compartment. Indeed, and in contrast to earlier observations, both Aire and Fezf2 are controlled by RANK signalling, with LTβR dispensable for Fezf2 expression [20]. Significantly, deletion of Fezf2 in TEC did not impair Aire expression, but resulted in an autoimmune phenotype, suggesting the functional importance of Fezf2 expression in the thymus for immune tolerance [19]. Therefore Aire and Fezf2 may function cooperatively, allowing for a more extensive TRA expression profile [21].

### MHCII^lo^CD80^lo^ mTEC^lo^

The mTEC^lo^ subset was originally described as an immature progenitor population that gave rise to mTEC^hi^ following RANK and CD40 stimulation. However, it has also been shown that mTEC^hi^ could revert back to a mTEC^lo^ phenotype following Aire expression [12, 22], suggesting that there is considerable heterogeneity within mTEC^lo^. This fits well with recent studies uncovering multiple and distinct mTEC subsets using single-cell RNA-Seq analysis [23–26], and further work is necessary to understand the development relationships and functionality of these subsets.

mTEC^lo^ that are generated from mTEC^hi^ are composed of multiple subsets. For example, they can begin to express markers including involucrin that are representative of terminally differentiated epithelial cells such as keratinocytes, which may further lead to the formation of structures that resemble Hassall’s corpuscles [27, 28]. These are readily identifiable in the human thymus, where they have been linked to DC activation and T-Reg generation [29]. In mice, though less pronounced in size and frequency, such structures have also been reported to play a role in DC activation that drives IFN-α production and T cell maturation [30]. An additional subset of mTEC^lo^, some of which are generated post Aire expression, are thymic tuft cells [23, 24], which are also seen in the human thymus [23, 27, 31, 32]. In mice (designated LTβR^TEC^), deletion of LTβR in TEC resulted in an absence of thymic tuft cells [33], which fits well with the importance of LTβR signalling during mTEC terminal maturation [34, 35]. Interestingly, analysis of tuft cells from different anatomical sites such as the colon, trachea, thymus, and bladder highlighted tissue-specific features of tuft cells and also showed that gut and thymus tuft cells had many similarities, including expression of IL-25, Dclk1, Trmp5, and Pou2f3 [36]. While the functional properties of thymic tuft cells have not yet been fully examined, conventional thymocyte development appears grossly normal in the absence of tuft cells [23, 24]. However, there is some evidence suggesting that through their expression of cell-type-specific molecules, tuft cells play a role in T cell tolerance mechanisms [24]. In addition, subtle defects in T-Reg [37], ILC2 [23], and iNKT cells [24, 33] have also been reported in tuft cell–deficient mice. Indeed, IL-25 expression by tuft cells was recently shown to influence IL-4-producing iNKT cells that regulate thymic DC [33]. Thus, emerging evidence suggests that tuft cells represent a functionally relevant mTEC subset in the thymus, which fits well with the idea that subsets within mTEC^lo^ are linked to thymus function.

Another striking example of the ability of mTEC^lo^ to influence T cell development is the identification of a subset that expresses the chemokine CCL21 [38]. Indeed, an essential requirement for CCL21 in the control of cortex to medulla migration of positively selected thymocytes identifies CCL21^+^ mTEC^lo^ as a critical, functionally mature mTEC^lo^ subset [39]. Interestingly, while the frequency of CCL21^+^ mTEC^lo^ is reduced in LTβR-deficient mice [38], their levels of CCL21 expression are not altered [33]. Thus, LTβR may control proliferation and/or survival of CCL21^+^ mTEC^lo^. However, the precursor-product relationships that give rise to CCL21^+^ mTEC^lo^ in relation to other mTEC^lo^ subsets, including tuft cells, are not well understood. The generation of CCL21-reporter mice [39] and the recent identification of CD104 as a cell surface marker of CCL21^+^-producing cells within mTEC^lo^ [33] should enable the direct isolation and further characterisation of these cells.

## Thymic dendritic cells and central tolerance

As well as the documented function of mTEC, DC can also be found in the thymus medulla from the late stages of embryogenesis, where they aid central tolerance induction [33, 40]. There are three populations of DC within the thymus, two conventional DC subsets (cDC1 and cDC2) and plasmacytoid DC (pDC) [41, 42].

### Intrathymic conventional dendritic cells 1

Recent findings indicate that conventional dendritic cells 1 (cDC1) develop from migrant pre-cDC progenitors that are distinct from the lymphoid lineage, and are recruited to the thymus via CCR7-CCL21 [43, 44]. Intrathymic cDC1 are typically localised within the medulla as they express the chemokine receptor XCR1 and mTEC produce its ligand XCL1 in an Aire-dependent manner [45]. The localisation of cDC1 near to mTEC is likely paramount to their function, as they specifically assist central tolerance by cross-presenting antigen via apoptotic material release or trogocytosis from Aire^+^ mTEC [46–48]. In a recent study, depletion of both cDC1 and mTEC resulted in the induction of organ-specific autoimmunity that was not seen in the selective absence of either mTEC or cDC1 alone, suggesting a cooperative functioning between these two populations for central tolerance [49]. Similarly, *aly/aly* mice with a point mutation in the NF-κB-inducing kinase (NIK) gene show disrupted mTEC and cDC1 numbers with subsequent peripheral autoimmunity, thus reinforcing the requirement for cross-talk between cDC1 and mTEC for negative selection [50].

### Thymic recruitment and function of extrathymic DC

cDC2 and pDC migrate into the thymus as mature cells and utilise cell adhesion molecules to gain thymic entry [51, 52]. cDC2 are recruited to the thymus through CCR2 expression with CCL8 (MCP-2) expressed by cTEC and surrounding blood vessels [53]. pDC migrate to the thymus via CCR9 expression and are likely attracted by CCL25-expressing TEC [52]. Additionally, pDC recruitment to the thymus may involve additional chemokine receptors and ligands. For example, pDC are receptive to CCR7 ligands in transwell migration assays [54]. Following thymic entry, cDC2 and pDC undergo extensive proliferation and upregulate expression of MHCII and CD80/CD86 priming them to interact with developing thymocytes and support tolerance induction [41]. Their ability to induce negative selection was demonstrated when OVA-pulsed DC were transferred intravenously into OT-II TCR transgenic mice, which resulted in the induction of thymocyte negative selection [41, 51]. In addition, as cDC2 are situated around blood vessels in the thymus, they are well placed to capture and present circulating antigens to support negative selection [53, 55, 56]. Furthermore, the capacity of the thymus to induce negative selection was shown to be increased by 4 weeks of age, and this correlated with a greater number of cDC2 migrating to the thymus with enhanced ability to present and process self-antigen [57].

### Thymic DC and T regulatory cell development

In addition to negative selection, DC have been associated with a role for induction of T-Reg development in the thymus [58]. This idea was originally controversial, as ablation of DC did not influence thymic T-Reg numbers [59, 60]. However, other studies have suggested that DC and mTEC play non-overlapping roles in the production of T-Reg with distinct TCR repertoires and that Batf3-dependent DC (cDC1) are crucial for T-Reg selection through acquisition and presentation of Aire-dependent antigens [61]. Interestingly, while these studies indicate the ability of DC to influence T-Reg development, they also suggest that some thymic DC may be more effective at supporting T-Reg generation than others. Whether this is due to differences in the intrathymic positioning of different DC subsets, or differences in their functional abilities as antigen-presenting cells, is not clear. Relevant to this, it is interesting to note that thymic DC have been reported as a source of IL-2 which is required for intrathymic T-Reg development [62] suggesting that the involvement of these cells in T-Reg generation extends beyond their provision of TCR ligands. Interestingly, however, IL-2 has also been shown to be produced by self-reactive CD4SP thymocytes [63], indicating that multiple cellular sources of IL-2 can influence T-Reg development in the thymus.

## Post-selection maturation of conventional αβT cells

In addition to mediating tolerance induction, the medulla also provides signals to ensure that conventional (i.e. CD25^−^Foxp3^−^CD1dtetramer^−^) CD4SP and CD8SP thymocytes undergo a differentiation programme prior to their exit from the thymus and entry into the circulation as recent thymic emigrants (RTE). As such, medullary located conventional SP thymocytes progress through a series of maturational stages that can be identified by expression of phenotypic markers, and acquisition of functional properties.

### Defining maturational states in conventional SP thymocytes

**Fig. 1:**
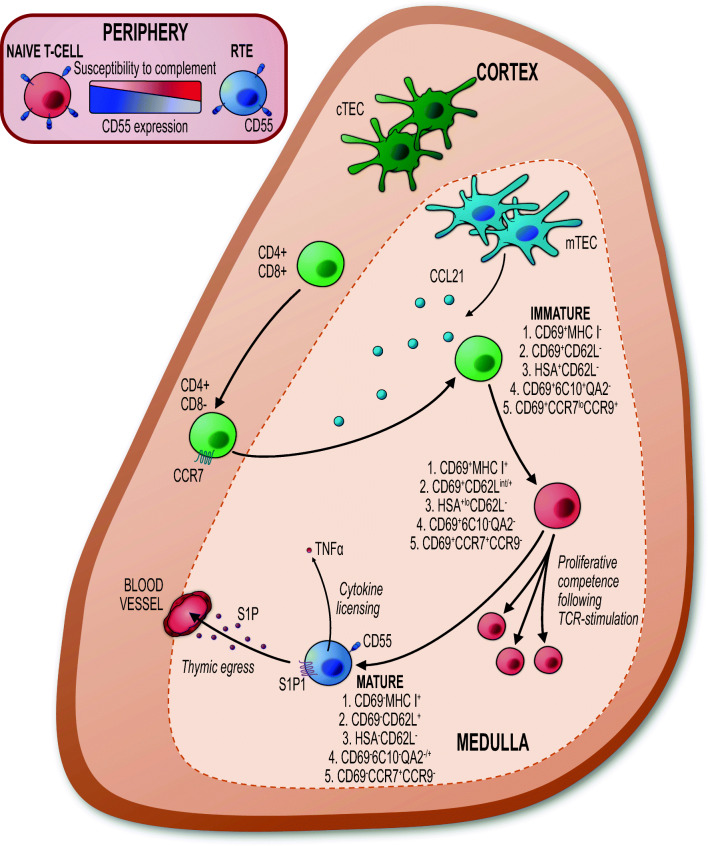
Phenotypic approaches to characterise post-selection maturation of conventional CD4SP αβT cells. CD4SP conventional thymocytes mature within the medulla, undergoing a number of phenotypic and functional changes. A consensus on which combination of phenotypic markers to use to identify each maturation state has not been reached, and different gating strategies have been used within the literature. Markers used, and how the expression of each changes through maturation, can be followed using the 1–5 numbering system for each of the different strategies that have been proposed. For example, #1 indicates the expression of CD69 and MHCI at each stage; immature CD69^+^MHCI^−^ ➔ CD69^+^MHCI^+^ ➔ mature CD69^−^MHCI^+^. Following initial maturation from an ‘immature’ state, CD4SP thymocytes gain the ability to proliferate in response to TCR ligation. Subsequent intrathymic maturation marks a transition when CD4SP thymocytes gain a series of functions: they upregulate CD55 to aid their protection from complement [83], become ‘cytokine licenced’ wherein they produce cytokines upon stimulation [74] and then expression of S1P1, which is essential for thymic egress, and entry into the periphery as recent thymic emigrants (RTE) [74, 81]. Although expression of CD55 is initiated in the thymus, its expression continues to increase in the periphery, reaching maximum levels on naïve αβT cells [83]

Classically, CD4 and CD8 are used to identify and study specific stages in T cell development in the thymus. For example, immature T cell progenitors reside within the CD4^−^CD8^−^ compartment which give rise to CD4^+^CD8^+^ cortical thymocytes. These then undergo selection events to generate CD4SP and CD8SP cells. Following identification of 4 main thymocyte populations based on CD4 and CD8 expression, later studies identified further heterogeneity within SP thymocytes. As discussed previously, this suggested different maturational states of SP thymocytes in the thymus [64]. Indeed, experiments involving BrdU pulse chase analysis provided strong evidence for distinct stages in the post-selection maturation of SP thymocytes [65]. Currently, many different parameters are also used to subdivide conventional SP thymocytes, in the hope of reaching a consensus on the maturational sequence of T cell development in the medulla [50, 66–69]. The use of Rag2GFP reporter mice has significantly helped in the accurate study of SP thymocyte maturation. For example, by separating bulk CD4SP thymocytes into 3 groups identified by differing Rag2GFP levels, initial studies showed that the most immature cells expressed high levels of CD69 and CD24. As these cells mature further, they downregulate CD69 and CD24 and upregulate CD62L and Qa2 [70, 71]. Thus, immature CD4SP thymocytes are CD24^+^CD69^+^CD62L^−^, while mature cells are CD24^−^CD69^−^CD62L^+^, and this transition is being accompanied by progressive loss of Rag2GFP levels. Additional cell surface markers including 6C10 and the chemokine receptors CCR7 and CCR9 also help define SP thymocytes and their precursor-product relationships [66, 68]. For example, using 6C10, CD69, and Qa2 expression, studies have shown that intrathymic transfer of immature CD69^+^6C10^+^Qa2^−^ (SP1) cells generated downstream CD69^+^6C10^−^Qa2^−^ (SP2), CD69^−^6C10^−^Qa2^−^ (SP3), and CD69^−^6C10^−^Qa2^+^ (SP4) populations [72, 73]. A summary of the various ways SP thymocytes have been subdivided is shown in Fig. 1.

While current studies frequently utilise combinations of the above markers in analysis of SP thymocytes, important observations have been made regarding the use of Qa2 [74]. Here, Qa2 levels in SP thymocytes were shown to be influenced by type I interferon signalling and so may not accurately reflect the maturational status of cell subsets [74]. Subsequently, an alternative method was described using CD69 and MHC I to identify 3 populations of SP thymocytes: SM (CD69^+^MHC I^−^), M1 (CD69^+^MHC I^+^), and M2 (CD69^−^MHC I^+^) [74]. The maturational relationship between these three populations was confirmed by Rag2GFP levels, as well as by identifying SM, M1, and M2 populations within previously established strategies, such as the SP1–4 or CCR7/CCR9 staining combinations [74]. Furthermore, the strength of this approach can be seen in the stratification of cell function to each subset. First, the switch to proliferation competence following TCR signalling was shown to occur at the M1 stage of development [74]. Second, maturation to M2 defines when thymocytes become ‘cytokine licenced’ and produce IFNγ and TNF-α upon TCR stimulation. Finally, the M2 stage marks the ability of SP thymocytes to exit the thymus [74]. The ability to accurately define progressive functional changes in SP thymocytes and the processes that control this rely on the ability to phenotypically define distinct developmental stages. As such, reaching a consensus on the panel of markers by researchers in the field, perhaps CD69/MHCI/CD62L/CCR7/CCR9 may aid progress in this area. It is also worth noting that much of the work described above relates primarily to CD4SP thymocytes, and while some marker combinations can help in subdividing CD8SP, there are clear differences in marker expression within the two lineages, suggesting that lineage-specific maturation markers may be required to accurately draw up a roadmap of both CD4SP and CD8SP thymocyte heterogeneity.

### Regulation of post-selection maturation

Consistent with its importance in T cell tolerance, mice that lack mTEC (e.g. *Relb*^*−/−*^ mice) or lack controllers of medullary access (e.g. *Ccr7*^*−/−*^ mice and *plt/plt* mice) develop autoimmunity [75, 76]. However, it is not clear to what extent mTEC may provide important signals that guide post-selection maturation. Indeed, the presence of phenotypically mature SP thymocytes in the thymus of *Relb*^*−/−*^ and *Ccr7*^*−/−*^ mice, and the presence of T cells in peripheral tissues, argues against an essential requirement for mTEC in post-selection maturation and thymic exit [39, 68, 77, 78]. However, it should be noted that mice with medullary defects have been reported to have defects in SP thymocytes, including a developmental block in CD4SP post-selection maturation in both *Relb*^−/−^ and *Aire*^−/−^ strains [72]. However, as these observations used Qa2 expression to measure maturation status, it is not clear whether there is indeed a maturation defect in these mice or whether altered Qa2 expression levels are a result of disruption of type I interferon signalling as discussed above [74]. Indeed, when *Relb*^*−/−*^ thymus lobes were grafted under the kidney capsule of wild-type (WT) hosts, CD4SP thymocyte maturation appeared normal, indicating that phenotypic CD4SP thymocyte maturation can occur independently of mTEC [68].

Interestingly, other studies provide evidence that the thymus medulla and/or mTEC may subtly influence the functional qualities of mature SP thymocytes and RTE. For example, when SP thymocytes of differing maturation states were isolated, intrathymically injected, and analysed 1 or 4 days post-injection, Jin et al. showed that cells spending longer in the thymus contained an increased proportion of IL-2, IL-4, and IL-10, as well as IFNγ-producing cells [73]. Thus, intrathymic dwell time may be proportional to functional acquisition, suggesting that the longer the time that thymocytes are able to spend within the thymus, the greater their functional capabilities. While the signalling events and mediators that control post-selection maturation are not well understood, examination of SM/M1/M2 SP thymocyte populations demonstrated that NF-κΒ- and IRF-regulated gene changes occurred later after positive selection [74]. Moreover, following deletion of *Tak1* (an important kinase in the NF-κΒ pathway), a block in the maturation at the SM stage was observed [74], with *Tak1* also protecting cells from TNF-induced death [74]. Similarly, a study investigating the role of NF-κΒ signalling in thymocyte development showed that deletion of the two subunits of the inhibitor of κΒ kinase (IKK) complex (IKK1 and IKK2) in thymocytes resulted in complete arrest of SP maturation at the immature CD24^hi^ stage [79]. Moreover, TNF signalling via TNFR1 was found to be required for activation of the NF-κΒ pathway, which was essential in protecting cells from TNF-mediated cell death [79]. Additional work from the same group confirmed this role for IKK in thymocyte survival and proliferation, but clarified that the essential role of the IKK activity is to repress RIPK1-kinase-dependent cell death by a mechanism which is independent of NF-kB [80]. Importantly, while some of the intracellular signalling cascades that are active in thymocytes to control post-selection maturation have been identified, the cell-cell interactions that trigger these signalling events are unclear.

### Thymus egress and SP thymocytes

The ability of SP thymocytes to exit the thymus is essential to establish the peripheral T cell pool. Egress competence is restricted to the mature fraction of thymocytes that express the sphingosine-1-phosphate (S1P) receptor 1 (S1PR1), which is essential for egress [81]. In relation to the control of this process, it has been reported that ligation of CCR2 on SP thymocytes induces activation of the transcription factor FOXO1-KLF2 axis which ultimately leads to expression of S1PR1, as well as enhancing S1P-induced chemotaxis itself [71, 73, 81, 82]. The importance of egress competency being limited to the most mature SP fraction may be beneficial as it may mean that only SP thymocytes that have undergone a full intrathymic maturation programme have the ability to leave the thymus, which may regulates the quality of the peripheral T cell pool. Relevant to this are studies examining patterns of expression of the cell surface marker CD55 in thymocyte subsets. For example, as CD55 protects peripheral T cells from complement-mediated cell death and high CD55 levels are limited to the most mature SP thymocytes [83], this indicates cells that have undergone full intrathymic maturation may have an enhanced ability to survive in the periphery [83]. Whether or not medullary residency, and contact with mTEC, is required for this process is not clear.

While the importance of S1PR1 expression by SP thymocytes in thymus emigration is well described, several differing models of emigration have been proposed. This is perhaps best represented by ‘conveyor belt’ and ‘lucky dip’ models of thymic exit [84]. In the former, exit of egress-competent thymocytes occurs in a developmentally controlled manner, suggesting that all SP thymocytes spend a similar period of time in the thymus. By contrast, the lucky dip model suggests that once SP thymocytes reach egress competence, they are eligible to leave the thymus irrespective of the amount of time they have spent in the medulla, and a consequence of this would be the export of cells of different ages. Recently, we used CD62L levels to identify subpopulations of CD62L^+^CD69^−^ M2 CD4SP thymocytes that we termed M2a, M2b, and M2c on the basis of their Rag2GFP levels, with M2a showing the highest GFP levels (as so are least mature) and M2c showing the lowest GFP levels (and so are the most mature). Using this approach, we showed that thymus egress follows an ordered regimen that supports a conveyor belt process rather than a random lucky dip. Thus, M2c thymocytes expressed the highest levels of S1PR1, perhaps giving the most mature cells a greater ability to exit the thymus, while cells within thymic perivascular spaces that represent sites of exit were enriched in M2c cells [85]. Therefore, while M2 (mature) thymocytes have the capacity to exit the thymus, within that population, there are cells with a greater ability to leave that is based on their age. What controls this ordered process of emigration is not known. However, it is interesting that while LTβR is an important regulator of thymic exit [67, 85], thymic emigration in LTβR-deficient mice still follows a conveyor belt mechanism [67, 85]. Further work is required to identify the microenvironmental signals that ensure thymus emigration is biased towards the most mature SP thymocytes.

As the cortico-medullary junction is a region containing blood vessels that represent sites of thymic exit, it is likely that TEC, and mTEC in particular, influence thymic egress. Indeed, in neonatal *Ccr7*^−/−^ mice where thymocyte migration to mTEC-derived chemokines CCL19 and CCL21 is abolished, thymus emigration is defective and results in intrathymic SP accumulation and a reduction in peripheral T cell numbers [78, 86]. That thymus emigration is not impaired in adult *Ccr7*^*−/−*^ mice [77, 86] indicates an interesting distinction to the neonate and suggests that the regulators of thymic exit, perhaps including a requirement mTEC themselves, are different at distinct stages of the life course. Additionally, in adult mice, mTEC can influence thymic egress by maintaining the S1P gradient essential for egress via their expression of lipid phosphate phosphatase (LPP3), an enzyme which dephosphorylates S1P to maintain S1P gradients [87]. In this study, deletion of LPP3 in K14^Cre^-positive TEC resulted in the intrathymic accumulation of mature SP thymocytes. It has also been shown that mTEC influence mature thymocyte egress indirectly, by regulating other lymphocyte populations that are present within the medulla. For example, LTβR-dependent regulation of iNKT cells regulates thymic egress [88, 89], while the influence of mTEC on DC via-NIK (NF-κΒ-inducing kinase) signalling is also required for normal thymocyte egress [50]. Again, much of the work carried out on thymus egress relates to CD4SP thymocytes and adult thymus. Given the different maturational pathways for CD4SP and CD8SP, and the differential requirement for CCR7 in neonatal but not adult thymus egress, further work is required to fully understand the role of the thymus medulla in controlling this process.

## iNKT cell development in the thymus medulla

### Redefining pathways in iNKT cell development

As is the case for conventional CD4SP and CD8SP thymocytes, iNKT cells arise from CD4^+^CD8^+^ thymocytes that reside in the cortex [90]. However, unlike these other lineages, iNKT cells do not recognise self-peptide/MHC complexes expressed by cTEC. Instead, interactions between CD4^+^CD8^+^ thymocytes involving an invariant αβTCR and lipid-presenting CD1d molecules initiate iNKT cell development [91]. Importantly, iNKT cells are an effective component of immune responses where they connect innate and adaptive arms of the immune system through their rapid production of cytokines that include IL-4, IFNγ, and IL-17 [6]. In early studies, cell surface markers such as CD24, NK1.1, and CD44 were used to suggest a linear sequence of iNKT cell development [92, 93]. More recently, use of CD1d/α-galactosylceramide (CD1d/αGC) tetramer reagents alongside transcription factor and cytokine expression revealed the development of multiple iNKT sublineages within the thymus. Similar to T helper cell nomenclature, three iNKT sublineages have been described: iNKT1 are T-bet^+^ and produce IFNγ, and iNKT2 are PLZF^+^ and produce IL-4, while iNKT17 are RORγt^+^ and produce IL-17 [94]. Although largely in agreement with the original description of these subsets, a subsequent study has also shown co-production of cytokines by iNKT cells following stimulation, in particular IL-4/IFNγ and IL-4/IL-17 by iNKT2 [95]. Interestingly, the composition of iNKT cells in the thymus of inbred strains of WT mice also differs. For example, C57Bl/6 mice have a prominent population of iNKT1, whereas iNKT in BALB/c mice are heavily dominated by iNKT2. Given that iNKT1 are defined by IFNγ expression while iNKT2 are defined by IL-4, this fits well with the respective type 1/type 2 immunity in these strains.

The identification of multiple iNKT sublineages in the thymus raises questions about the developmental pathways that give rise to these cells. Importantly, Lee et al. identified CCR7 expression as a potential iNKT cell progenitor marker [96]. Analysis of iNKT cell development in Rag2GFP mice showed that in contrast to CCR7^−^ iNKT cells, CCR7^+^ iNKT cells retain some GFP expression, indicative of an early stage in iNKT cell development [97]. Importantly, CCR7^+^Rag2GFP^+^ iNKT cells gave rise to iNKT1, iNKT2, and iNKT17 cells following their intrathymic transfer, providing direct evidence they represent a common iNKT progenitor. Interestingly, CCR7^+^Rag2GFP^*+*^ iNKT progenitors express markers previously shown to be present early in iNKT cell development such as Plzf and Lef1 [97]. However, they lack expression of transcription factors and cytokines that indicate more mature iNKT cells such as RORγ, T-bet, and IL-4 [97]. Interestingly, CCR7^+^ iNKT cell progenitors can also exit the thymus and continue their development towards iNKT1, iNKT2, and iNKT17 extrathymically [97]. Why some CCR7^+^ iNKT progenitors choose to complete their maturation intrathymically as opposed to maturing extrathymically is not clear.

### Intrathymic control of iNKT lineage heterogeneity

**Fig. 2:**
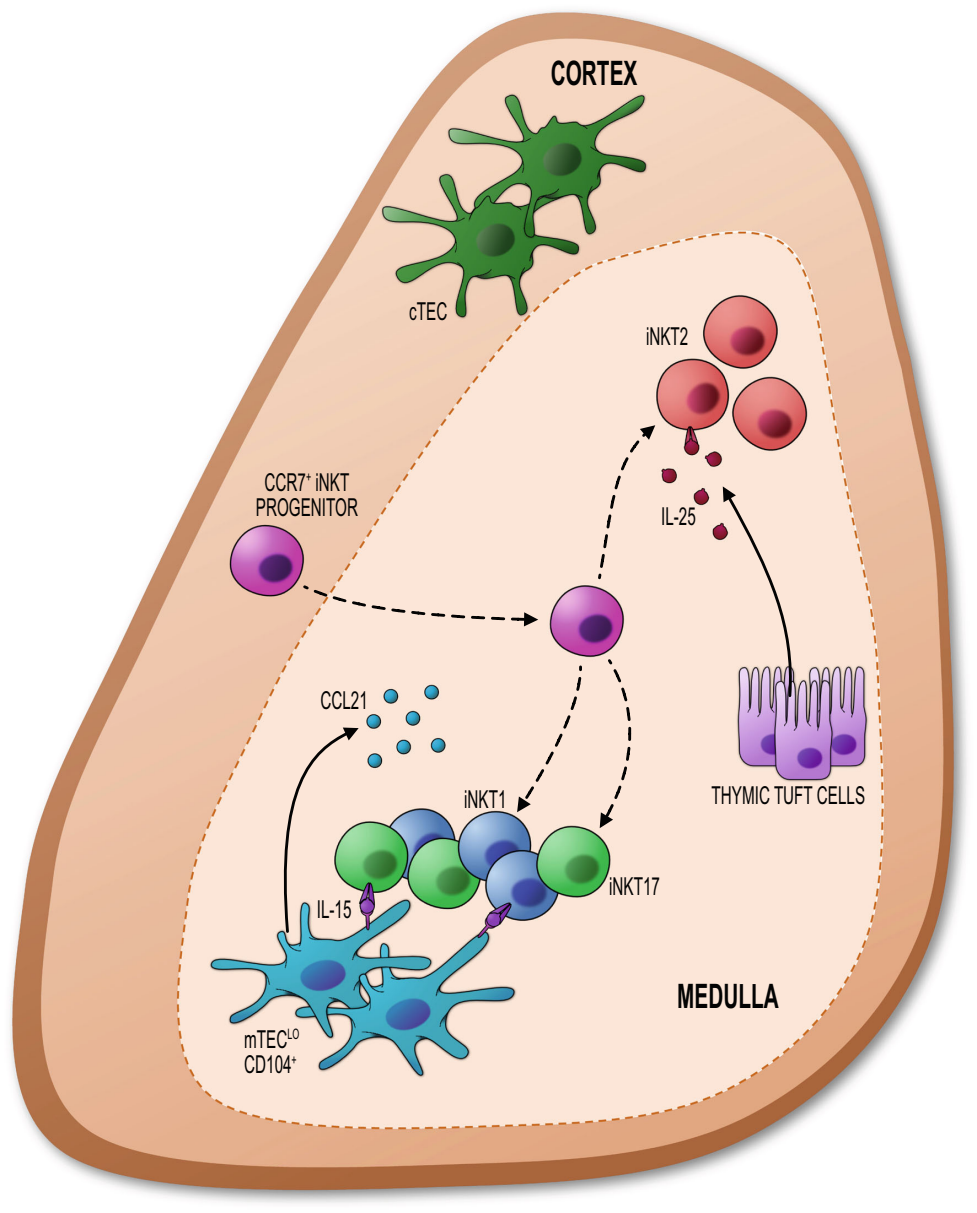
Regulation of intrathymic iNKT cell development by distinct populations of mTEC. CCR7^+^ iNKT cell progenitors migrate into the thymus medulla in a CCR7-dependant manner. Here, perhaps under the influence of medullary stroma, they can undergo further differentiation to generate iNKT1, iNKT2, and iNKT17 subsets. The IL15Rα-mediated transpresentation of IL-15 by CD104^+^ mTEC^lo^ influences the intrathymic availability of both iNKT1 and iNKT17. In contrast, iNKT2 numbers in the thymus are regulated by IL-25, a selective product of thymic tuft cells that are generated during mTEC terminal differentiation

Although initial stages of iNKT cell development require interactions between DP thymocytes in the cortex, analysis of *Relb*^*−/−*^ mice demonstrates a clear requirement for mTEC in their downstream maturation [34] (Fig. 2). Consistent with this, iNKT cells are primarily located within the thymus medulla in the adult mouse [98]. This positioning is partially dependent on CCR7, as mixed chimeras using WT and *Ccr7*^*−/−*^ bone marrow resulted in the mispositioning of some *Ccr7*^*−/−*^ iNKT cells to the thymic cortex [97]. Studies that have highlighted the significance of the thymus medulla for iNKT cells have raised important questions about factors produced by mTEC, and whether particular mTEC subsets influence the development of individual iNKT subsets.

The identification of multiple intrathymic iNKT subsets, together with an expansion of our understanding of mTEC heterogeneity, has allowed further examination of links between mTEC and iNKT cells. In a recent study, LTβR^TEC^ mice, which specifically lack expression of LTβR by TEC, have been used to determine the effects of disrupted medullary environments on iNKT cell development [33]. This study showed that LTβR controls the frequency of CD104^+^CCL21^+^ mTEC^lo^ [33, 38] that are capable of IL-15 transpresentation, which has been linked to the proliferation and survival of iNKT cells [34, 99]. Interestingly, CD122 (a subunit of IL-15 receptor) is expressed by all thymic iNKT cells with the highest levels being expressed by iNKT1 [33, 94], and in vivo administration of IL-15/IL-15Rα complexes increased iNKT1 and iNKT17 in LTβR^TEC^ mice [33]. Importantly, and in contrast to in vivo experiments, in vitro IL-15/IL-15Rα treatment increased numbers of iNKT1 only, suggesting that iNKT17 require additional factors that are present within the thymus but absent in vitro [33]. Interestingly, iNKT17 show evidence of recent TCR signalling in Nur77GFP mice [100], suggesting that IL-15 transpresentation by mTEC, plus further interactions with CD1d-expressing cells in the thymus, could be required for iNKT17 development. Other factors have been shown to influence the development of iNKT17 including TGFβ [101], and the serine protease SerpinB1 [95]. The mechanisms behind this involvement, and any potential synergy between these pathways, are unknown.

Further evidence surrounding the importance of mTEC in iNKT cell development comes from analysis of thymic tuft cells that represent a terminally differentiated mTEC subset [23, 24]. Interestingly, initial descriptions of iNKT cell development in tuft cell–deficient *Pou2f3*^−/−^ mice reported reductions in all 3 iNKT1, iNKT2, and iNKT17 sublineages [24]. However, a selective reduction in iNKT2 in the thymus of *Pou2f3*^−/−^ mice was reported [33], with numbers of iNKT1 and iNKT17 remaining unaltered. That iNKT cell subset frequencies are background strain dependent may provide an explanation for this discrepancy. However, that both studies demonstrate a role for tuft cells in iNKT2 cell development is consistent with a specific reduction in iNKT2 in *Il25*^−/−^ mice, which indicates that the importance of tuft cells in iNKT2 cell development may be explained at least in part by their selective production of IL-25 [23, 33]. This finding fits well with selective expression of IL-25 receptor by iNKT2 but not iNKT1 and iNKT17 [24]. Collectively, these observations suggest that multiple epithelial cell subsets reside within the mTEC^lo^ population, which are functionally specialised to support the development of specific iNKT lineages.

### Thymus emigration and retention of iNKT cells

While intrathymic CCR7^+^ iNKT cells have been shown to include cells with progenitor potential [97], these cells are also significantly enriched within the pool of iNKT RTE that are present in peripheral tissues [97]. Thus, these cells may represent the predominant iNKT cell subset that exits the thymus [97] such that CCR7^+^ iNKT progenitors play an important role in establishing the peripheral iNKT cell pool [97]. While expression of CCR7 and RagGFP have been investigated as potential defining markers of iNKT RTE, characterisation of RagGFP^+^ iNKT shows an enrichment of CCR7^+^ cells in the lymph node but not the spleen, suggesting that while CCR7 is a RTE useful marker, it may not be a universal iNKT RTE marker [102]. In agreement with this, another study used intrathymic biotin injection to label a cohort of thymic iNKT and subsequently track these cells as RTE in the periphery. Interestingly, although CCR7^+^ cells were enriched within the labelled fraction, only around 50% of iNKT RTE expressed CCR7 [97].

An additional challenge to studying iNKT RTE is that, unlike the case for conventional thymocytes, the majority of iNKT cells in the thymus are RagGFP negative. Thus, in addition to RagGFP^+^ iNKT cells, the thymus may also control the exit of RagGFP^−^ iNKT cell subsets, which also either act as progenitors or represent cells that undergo lineage commitment intrathymically. In agreement with the idea that iNKT RTE undergo differentiation in the periphery, comparison of the TCR repertoire highlights significant differences between RagGFP^+^ iNKT RTE and RagGFP^−^ iNKT [102]. Moreover, the TCR repertoire of iNKT cells is distinct in different peripheral tissues, showing that the clonal expansion of iNKT cells is determined by their anatomical location [102]. The frequency of RagGFP^+^ iNKT RTE also differs between tissues, with a high proportion of RagGFP^+^ iNKT RTE in mesenteric lymph nodes (mLN), suggesting either a differential rate of RTE homing to certain tissues, or a more rapid turnover of RagGFP^+^ iNKT, resulting in a decline in RagGFP expression [102].

Importantly, although some iNKT RTE express CCR7 and RagGFP, whether populations of intrathymically generated iNKT1, iNKT2, and iNKT17 are permanent thymic residents or can also contribute to the peripheral pool of iNKT remains unclear [95, 97]. Relevant to this, studies using multiple experimental approaches including thymus grafting, parabiosis, and measurement of Rag2GFP expression have shown that the majority of iNKT cells are retained within the thymus and are not under constant replenishment [97, 103]. The chemokine receptor CXCR3 has been identified as an important factor involved in the thymic retention of iNKT cells. Analysis of CXCR3-deficient mice revealed reduced frequencies of iNKT cells in the thymus and an increased frequency in the blood, suggesting its involvement in controlling the balance of iNKT cells in the thymus and peripheral circulation [104]. Furthermore, intrathymic injection of FITC into WT and *Cxcr3*^*−/−*^ mice showed increased frequencies of FITC^+^ iNKT RTE in the absence of CXCR3 [104], thus illustrating this chemokine receptor acts to prevent iNKT cell emigration from the thymus. In addition, CXCR3 is expressed most highly by iNKT1 [95], which may explain why despite their expression of S1PR1, very few iNKT1 leave the thymus.

That the thymus retains a large proportion of the iNKT cells it generates raises intriguing questions over what function these cells might possess. iNKT cells are known to be steady-state producers of cytokines, including IFNγ, IL-17, and IL-4. However, our understanding of the functional significance of these cytokines in the thymus is largely limited to IL-4. Using cell-specific deletion of CD1d, Wang et al. showed that macrophages present endogenous ligands that trigger the TCR of iNKT2 cells, resulting in IL-4 production [100]. Intrathymic production of IL-4 by iNKT cells is required for the generation of innate memory-like CD8^+^ thymocytes, which are characterised by their expression of Eomesodermin. Such CD8^+^ thymocytes are reduced to near-absent levels in *Il4ra*^−/−^ and *Cd1d*^*−/−*^ mice [105] and are also found in much lower frequencies in C57Bl/6 thymus, compared to BALB/c thymus, where IL4-producing iNKT2 are the major population of iNKT cells [94]. In addition, iNKT2 have been identified as a key regulator of thymocyte emigration, as mice deficient in either iNKT cells, or components of the IL-4 signalling pathway, show large perivascular accumulations of mature CD4SP thymocytes coupled with a reduction in their levels of Rag2GFP [105]. Interestingly, this study also showed that thymocyte emigration was further hindered when *Il4ra*^−/−^ mice were treated with FTY720, an agonist for S1PR1 that downregulates this receptor and so prevents S1P-mediated thymus emigration. Importantly, this finding indicates that the mechanism by which IL-4 promotes thymocyte egress is distinct from S1P [105]. As IL-4 is a signature cytokine of iNKT2, these findings suggest that iNKT2 cells represent at least some of the iNKT cells that are retained within the thymus, and suggest a functional explanation for their thymus residency. While the possible functional importance of the intrathymic retention of iNKT cells requires further study, that thymic iNKT cells regulate both DC and mTEC suggests that individual iNKT subsets play additional roles in governing multiple aspects of thymus medulla development and function [33, 34, 94, 97].

## Conclusions

The importance of intrathymic microenvironments to foster and shape the αβTCR repertoire is now well established. Currently, properties of the medullary areas of the thymus are known to significantly extend beyond their capacity to induce central tolerance in conventional αβT cells. While additional support for these cells from mTEC and medulla areas includes phases of post-selection maturation and emigration into peripheral tissues, both of these aspects of medulla function remain incompletely understood. Importantly, that the role of the medulla also extends to the generation of self-regulating Foxp3^+^ T cells and CD1d-restricted iNKT cells, further emphasises how this site influences the diversity of intrathymic T cell development. By gaining a better understanding of how new heterogeneity in mTEC populations relates to the diversity of medulla functions, future research will provide insight into the development, function, and eventually the therapeutic manipulation of thymic microenvironments.
